# Lymphoma Induced Chylothorax Complicated by Disseminated Histoplasmosis and Esophagopericardial Fistula

**DOI:** 10.1002/ccr3.70886

**Published:** 2025-09-11

**Authors:** Bo Liu, Kristina Aleksoniene, Sapna R. Patel, Patrick C. McKillion, Michael S. Wang

**Affiliations:** ^1^ Department of Medicine Corewell Health Southwest Saint Joseph Michigan USA; ^2^ Osteopathic Medical Specialties Michigan State University College of Osteopathic Medicine East Lansing Michigan USA; ^3^ Division of Hematology and Oncology Bronson Methodist Medical Center Battle Creek Michigan USA; ^4^ Division of Critical Care Medicine Evangelical Community Hospital Lewisburg Pennsylvania USA; ^5^ Department of Medicine Central Michigan University School of Medicine Saginaw Michigan USA

**Keywords:** chylothorax, disseminated histoplasmosis, esophago‐pericardial fistula, non‐Hodgkin's lymphoma, pleural effusion

## Abstract

This case report highlights the need to consider both infection and malignancy in the differential of a chylothorax, particularly in an immunosuppressed patient. The case demonstrates the challenges in treating a chylothorax, both in terms of appropriate drainage and the necessity to maintain a low‐fat diet. The case also presented challenges in treating histoplasmosis, both in terms of renal effects of amphotericin B and the effect of a low‐fat diet on an oral antifungal agent. Although infection may be controlled with appropriate antifungal agents, the underlying hematologic malignancy must be controlled in order to successfully treat the patient.

**Summary**

- Diagnosis of the etiology of a chylothorax is complex, with the differential including infection, malignancy, and trauma.
- Conservative treatment includes addressing the underlying etiology, a low‐fat diet, appropriate medications, and chest tube drainage.
- Treating histoplasmosis in an immunosuppressed patient is challenging, particularly when a patient is unable to consume a high‐fat diet.

## 1 Introduction

Chylothorax is defined as the accumulation of chyle in the pleural space. Diagnosis is confirmed by a triglyceride concentration greater than 110 mg/dL and a predominance of lymphocytes (> 70% of total nucleated cell count) [1]. The etiologies of chylothorax are generally classified as traumatic or non‐traumatic [1]. Traumatic etiologies may include non‐penetrating trauma, including rapid acceleration–deceleration injuries or penetrating trauma [2]. Surgical injuries to the thoracic duct have been reported as complications of chest and neck surgeries, particularly esophagectomies [1, 2]. In addition, chylous leaks due to fistulas have also been reported after neck dissections, particularly in patients with thyroid cancer [3, 4]. Non‐traumatic causes can be further subdivided into malignant and non‐malignant origins. Among the malignancies associated with a chylothorax, lymphoma is the most common cause. Lung cancer, mediastinal tumors, chronic lymphocytic leukemia, Kaposi sarcoma, multiple myeloma, and metastatic cancers are also significant contributors [1]. A multicenter study [2] reported that 37% of chylothorax cases were caused by lymphoma. Another series [1] attributed 11% to lymphoma and 48% to surgical trauma. Interestingly, there has only been one pediatric case linking chylothorax directly to histoplasmosis [5].

*Histoplasma capsulatum* is a fungal pathogen endemic to North and Latin America [6, 7], particularly in the Mississippi and Ohio River valley [6, 7]. The clinical spectrum of histoplasmosis varies widely. Most individuals are either asymptomatic or experience mild, self‐limited symptoms [6, 7, 8]. When symptomatic, histoplasmosis most commonly manifests as a pulmonary infection [6, 7, 8, 9]. The presentation can be either as an acute pulmonary histoplasmosis, which is often self‐resolving [6, 7, 8, 9] or a chronic cavitary pneumonia requiring antifungal therapy [6, 7, 8]. Disseminated histoplasmosis is more common in the immunocompromised population. Other rare manifestations include mediastinal granuloma and fibrosis [7, 8].

Pleural involvement in histoplasmosis is uncommon [8, 9, 10, 11] and is thought to arise from a caseous subpleural focus leaking into the pleural space [9, 11]. Due to the rarity of pleural disease, the role of immunosuppression remains uncertain as both immunocompetent and immunocompromised cases have been reported [8, 9, 10, 11, 12]. Pericarditis and pericardial effusions secondary to histoplasmosis are similarly rare [13, 14]. Lymphoma is also an established cause of a pericardial effusion [15].

We report a case of a patient with non‐Hodgkin follicular lymphoma complicated by pleural and disseminated histoplasmosis. His course was further complicated by the formation of an esophageal‐pericardial fistula.

## 2 Case Report/Examination

**FIGURE 1:**
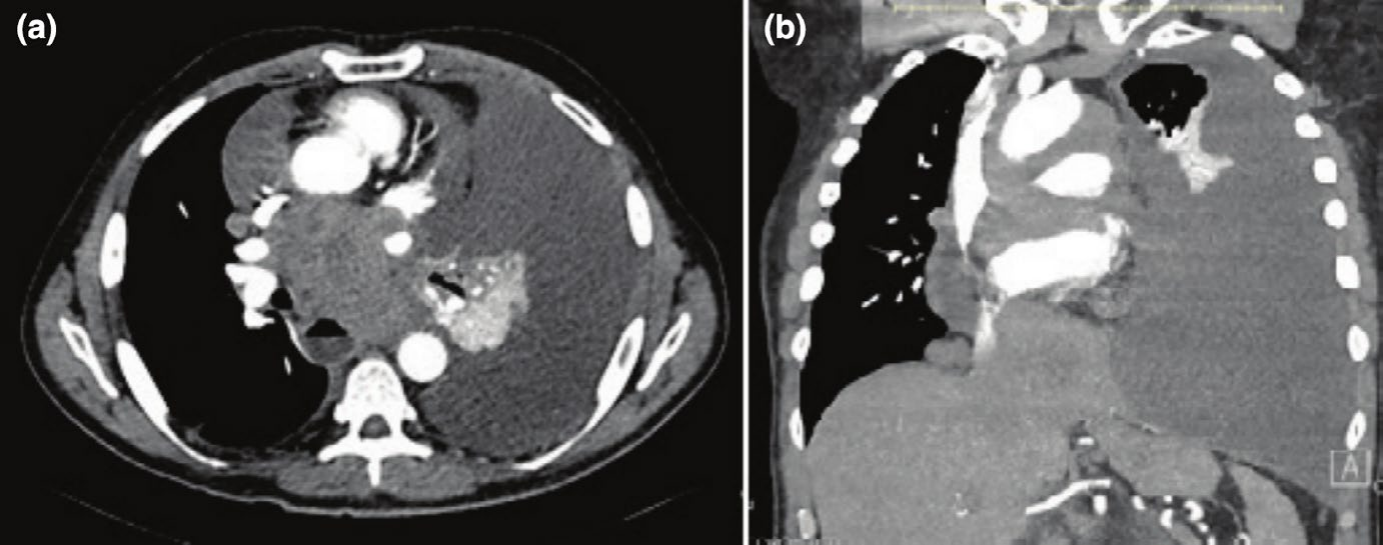
Cross sectional (a) and coronal (b) views of pleural effusion.

A 67‐year‐old male with a history of non‐Hodgkin lymphoma, in remission for one year post‐chemotherapy, presented with progressive dysphagia. Chest radiographs and PET/CT scans revealed a new left pleural effusion. Diagnostic and therapeutic thoracentesis confirmed the presence of a chylothorax based on fluid analysis. Following one cycle of CHOP and Obinutuzumab, the patient developed fever, malaise, and lightheadedness and required emergent transfer to the emergency department (ED) from the infusion center. The CBC demonstrated a WBC of 0.0 10^3^/μL and Platelets of 17.0 10^3^/μL (Table 1). On ED arrival, the patient was hypotensive, tachycardic, and had a low‐grade fever. Physical exam demonstrated decreased breath sounds in the left lower lung field. His room air pulse oximetry was 97%. Repeat Chest X‐ray (CXR) (Figure 1a) and CTA chest (Figure 1b) demonstrated the recurrence of a large left pleural effusion in addition to a mediastinal effusion. There was a significant decrease in the size of the posterior mediastinal and subcarinal masses.

**TABLE 1:** Trend of laboratory results and chest tube output.

	Admit^a^	Hospital Day#3^b^	Hospital Day #7	Hospital Day #12^c^	Hospital Day #14	Hospital Day #20^d^	Hospital Day #30^e^
WBC‐cells 10^3^/μL (4.5–11.0)	0.0	0.4	2.7	25.8	25.4	6.9	3.2
HGb g/dL (14.0–18.0)	10.2	9.8	7.9	8.4	8.7	7.4	7.5
Plt 10^3^/μL (140–440)	17	14	15	52	88	126	137
Creatinine mg/dL (0.7–1.2)	0.8	1.6	1.4	1.4	1.33	0.8	1.1
Chest tube output (cc)		780	520	20	1630	260	170

^a^ 39 days after initial thoracentesis, patient with chest tube placed, filgastrim started.

^b^ Amphotericin B changed to voriconazole.

^c^ New chest tube placed.

^d^ Re‐started amphotericin B.

^e^ Day of discharge.

## 3 Differential Diagnosis, Investigations, and Treatment

**FIGURE 2:**
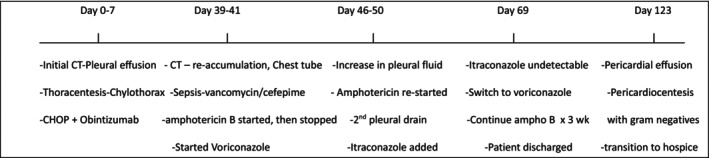
Timeline.

The patient was admitted to the Intensive Care Unit with septic shock and febrile neutropenia (Figure 2). A chest tube thoracostomy was performed to drain the pleural fluid. Fluid analysis re‐demonstrated the chylothorax. Empiric antimicrobial therapy included cefepime and vancomycin for septic shock. Pegfilgrastim was administered for febrile neutropenia. Midodrine was administered as therapy for both the hypotension and chylothorax.

The initial differential diagnoses included tumor obstruction of the thoracic duct and both bacterial and opportunistic infections (Table 2). Disseminated histoplasmosis was suspected based on the identification of intracellular yeast forms in the manual differential of the CBC. Although fungal cultures, immunodiffusion, and complement fixation tests were negative, a urine histoplasma antigen test returned positive. A pleural fluid culture ultimately grew an unidentifiable mold. Empiric antifungal therapy was initiated with liposomal amphotericin B. Due to nephrotoxicity and an acute kidney injury, both the liposomal amphotericin B and vancomycin were discontinued. Antifungal therapy was switched to oral voriconazole. The chest tube drainage was initially 800 mL/day for 5–6 days and gradually decreased to 0 mL/day by day 10. Repeat CT chest continued to demonstrate a large left pleural effusion that was separate from the existing left chest tube catheter. He underwent a second chest tube thoracostomy with an increase in pleural fluid drainage to 750 mL/day. Pleurodesis was considered by thoracic Surgery but was aborted due to the high surgical risk and persistent hypotension. Antifungal therapy was modified to three times per week amphotericin B and daily oral voriconazole. The chylothorax eventually resolved with chest tube catheter drainage after 1 month of hospitalization. He was transitioned to oral itraconazole in anticipation of discharge, but his itraconazole levels were undetectable due to a low‐fat diet impacting drug absorption. He was subsequently discharged home on oral voriconazole, planned for at least a 12‐month course, and a 3‐week course of parenteral amphotericin B.

**TABLE 2:** Differential diagnosis of chylothorax and pleural effusion.

Tumor obstruction of thoracic duct
Malignant effusion
Fistula secondary to tumor invasion
Bacterial empyema
Fungal infection
Mycobacterial infection

## 4 Outcome and Follow‐Up

**FIGURE 3:**
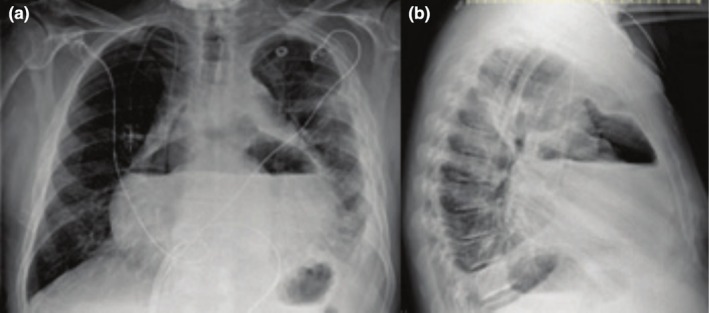
Chest X‐ray—Frontal (a) and lateral (b) views of pericardial effusion.

Chemotherapy was resumed after the completion of amphotericin B. Four months after antifungal therapy and three weeks after his second chemotherapy cycle, he presented to the ED with septic shock. CXR demonstrated pneumohydropericarditis (Figure 3). A pericardial window was placed with drainage of 1 L of purulent milky yellow fluid. Pericardial fluid culture grew a *Providencia* species, *Streptococcus gordonii* , *Streptococcus anginosus*, and *Streptococcus constellatus* . CT Chest post‐drainage confirmed the presence of an esophagopericardial fistula. His clinical condition deteriorated with mediastinitis‐induced septic shock with multiple organ system failure. The family decided to change goals of care with the withdrawal of life support therapy. He was transitioned to comfort care before subsequently expiring.

## 5 Discussion

Histoplasmosis can present as disseminated disease, particularly in the immunocompromised population [7, 8]. Disseminated histoplasmosis is commonly diagnosed by a positive urine histoplasma antigen or visualization of yeast forms on a peripheral blood smear [8, 16, 17, 18]. In our case, the patient presented with intracellular yeast forms identified during a manual CBC differential and a positive urine histoplasma antigen confirming the diagnosis of disseminated histoplasmosis.

The etiology of the patient's chylothorax was initially attributed to the non‐Hodgkin's lymphoma. The recurrence of his pleural effusion despite appropriate chemotherapy raised suspicion for an additional infectious cause. Pleural fungal cultures did grow a mold, but the organism was not identifiable. The positive urine histoplasma antigen test combined with the findings of intracellular yeast inferred a diagnosis of a pleural histoplasmosis infection. This case highlights the complexity of diagnosing the underlying etiology in immunocompromised patients with overlapping malignancy and infection. Although a malignant effusion was high in the differential, we speculate that it was more likely that the tumor obstructed the thoracic duct, leading to the chylothorax and providing a media for the histoplasma organism to grow. His immunosuppression raised the level of suspicion for an invasive fungal infection, which prompted early initiation of antifungals.

### 5.1 Diagnostic Management

Pleural histoplasmosis is an uncommon manifestation of the disease [8, 9, 10, 11, 12]. The mechanism of chylothorax from lymphoma involves direct disruption and/or compressive obstruction of the thoracic duct resulting in dysfunctional reverse flow of chyle toward the lung [1, 2]. Etiologic investigation commonly begins with a Chest CT scan to evaluate for mediastinal masses and evaluation of the thoracic duct patency [19]. In our patient, histoplasmosis was not suspected until the recurrence of his effusion. We hypothesize that the initial effusion and chylothorax were secondary to obstruction of the thoracic duct by his non‐Hodgkin's lymphoma. The patient's risk for pleural histoplasmosis was enhanced by the presence of the chylothorax and his immunosuppression. It is unclear whether the histoplasmosis was present upon his initial thoracentesis. The unidentified mold grown on the repeat pleural fluid fungal culture led us to suspect that pleural histoplasmosis was present at the time of the repeat thoracentesis.

Additional diagnostic studies for lymphatic imaging include lymphangiography, lymphoscintigraphy, and MRI lymphangiography [20]. These are obtained to evaluate for a thoracic duct tear, anomalous thoracic duct anatomy, and an unknown source of chyle leak where an intervention is planned [21, 22]. In our case, additional testing to perform embolization of the cisterna chyli was considered due to the persistent chylothorax requiring a second chest tube thoracostomy. However, this was not performed since the patient's chylothorax soon started to decrease in volume and subsequently resolved.

### 5.2 Management

Management may include conservative measures including dietary modifications, fluid and electrolyte replacement, medications (somatostatin or octreotide), or pleural drainage. As this chylothorax was recurrent, clinicians should be cognizant of the potential for relapse, and surveillance imaging could be considered for earlier intervention. If conservative management is unsuccessful, then surgical or percutaneous repair may be necessary [2, 23, 24, 25, 26]. High output chylothorax (> 1 L chyle/day) indicates thoracic duct disruption, common in post‐surgical settings [27]. Low output chylothorax treatment involves continuous pleural drainage with a chest tube thoracostomy, but is associated with long‐term loss of protein, lymphocytes, and immunoglobulins [27]. Patients usually require an oral or enteral high protein, low‐fat diet (< 10 g/day) supplemented with fat‐soluble vitamins and a parenteral fat emulsion [27, 28]. Our patient received aggressive nutritional therapy as outlined above. Octreotide has been used in chylothorax management by inhibiting the absorption of chyle from the intestine [29]. Midodrine has been demonstrated to induce a vasoconstriction effect on the lymphatic system in the setting of chylothorax [30]. Oral midodrine was administered throughout our patient's hospital course due to both the chylothorax and hypotension.

Interventional radiology modalities include thoracic duct embolization (TDE) and thoracic duct disruption (TDD) [2, 23, 24, 25]. TDE is performed by injecting contrast into the thoracic duct to find the leak and sealing it with embolized coils and glue [23, 24, 25]. TDD, which is a misnomer for the retroperitoneal lymphatics that are disrupted and macerated, rather than the thoracic duct itself, is performed by macerating the retroperitoneal lymphatics with multiple needle passes under fluoroscopy [23, 24, 25]. It is hypothesized that the viscosity of the iodinated oil contrast agent, the lymph leak from the punctures, and the small retroperitoneal hematoma may diminish lymph flow enough to close the leak [23, 24, 25]. Thoracic duct ligation is performed via open thoracotomy or video‐assisted thoracoscopy and has a success rate > 90% for postoperative chylothoraces [23, 24, 25]. Pleuro‐peritoneal shunting is a less invasive surgical approach, but is contraindicated in patients with concurrent chylous ascites and also may increase the risk of infection [24]. Pleurodesis via chest tube is a non‐surgical alternative in patients who are not surgical candidates [23, 24]. There are no guidelines regarding the timing of performing chemical or surgical pleurodesis or percutaneous TDE/TDD [2, 23, 24]. Intermittent thoracentesis or an indwelling pleural catheter are indicated for pleural effusions that accumulate at a rate < 50 mL/day [30].

According to the 2007 IDSA Guidelines on the treatment for histoplasmosis, amphotericin B is recommended for severe disease [31]. Given our patient's presentation with histoplasmosis dissemination and a recurrent pleural effusion requiring multiple chest tube thoracostomies, antifungal therapy was initiated with amphotericin B, which was consistent with guidelines [31]. Itraconazole is recommended as an oral stepdown agent [31]. Itraconazole absorption is enhanced by food absorption [32], but our patient required NPO status due to his chylothorax. In addition to re‐starting amphotericin at a lower dose, we opted to start voriconazole in place of itraconazole, as it is a recommended antifungal therapy for neutropenic fever per IDSA guidelines [33], and concerns about the absorption of itraconazole due to the need for a low fat diet [32]. Enhancing itraconazole absorption in the context of a low fat diet was not feasible, as itraconazole is lipophilic [32], thus any attempt to add fat into his diet likely would have been detrimental to the treatment of his chylothorax. Voriconazole has been successfully used in patients with both pulmonary and disseminated histoplasmosis [34, 35, 36, 37]. Although we believe that our patient had a good response, the overall non‐inferiority of voriconazole as compared to itraconazole has not been established [36]. An observational study found that the mortality of patients with histoplasmosis was higher with voriconazole as opposed to itraconazole [37]. Further investigation and studies are needed to investigate the use of voriconazole in this setting. Although studies are limited, there are studies that support the use of posaconazole for the treatment of histoplasmosis [38, 39]. For mild to moderate disease requiring treatment, 6–12 weeks of therapy is recommended [31]. For cavitary disease or disseminated disease, which is consistent with our patient, at least 12 months of therapy is recommended [31].

Malignancies, including lymphoma, have been established as a cause of pericardial effusions [15]. Although histoplasmosis could be an etiology for the pericardial effusion in our patient, pericardial fluid cultures and cytology were negative for histoplasmosis. This made the diagnosis less likely, especially while receiving guideline‐directed therapy for histoplasmosis. Based on his clinical presentation and the finding of enteric pathogens in the pericardial fluid culture, we speculate that the esophageal‐pericardial fistula was secondary to the progression of his lymphoma. It is likely that the tumor invaded through his esophagus into the pericardium, resulting in the fistula and subsequent pneumohydropericarditis. Esophagopericardial fistulas are rare, but have been reported in patients with upper GI malignancies [40].

The complexities of this case required coordination of multiple specialties and a multidisciplinary approach. Different aspects included ICU level support, managing antifungal therapy, chest tube support, parenteral nutrition, and chemotherapy regimens. This case also highlights the significant morbidity and mortality related to the patient's underlying non‐Hodgkin's lymphoma.

## 6 Conclusion

Chylothorax secondary to non‐Hodgkin lymphoma can predispose patients to disseminated histoplasmosis. Management requires adequate pleural drainage, low‐fat diet, and long‐term antifungal therapy. The absorption of antifungals such as itraconazole can be complicated by the low‐fat diet.

## Author Contributions

**Bo Liu:** methodology, writing – original draft. **Kristina Aleksoniene:** investigation, methodology. **Sapna R. Patel:** investigation, methodology. **Patrick C. McKillion:** methodology, supervision, writing – review and editing. **Michael S. Wang:** methodology, supervision, writing – review and editing.

## Ethics Statement

The authors have nothing to report.

## Consent

Written informed consent was obtained from the patient for the publication of this case report and any accompanying images.

## Conflicts of Interest

The authors declare no conflicts of interest.

## Data Availability

The data that support the findings of this study are available on request from the corresponding author. The data are not publicly available due to privacy or ethical restrictions.
